# Acoustic Radiation Force Impulse Imaging for Differentiation of Thyroid Nodules

**DOI:** 10.1371/journal.pone.0042735

**Published:** 2012-08-29

**Authors:** Joerg Bojunga, Nina Dauth, Christian Berner, Gesine Meyer, Katharina Holzer, Lisa Voelkl, Eva Herrmann, Hartmut Schroeter, Stefan Zeuzem, Mireen Friedrich-Rust

**Affiliations:** 1 Department of Internal Medicine 1, J.W. Goethe-University Hospital, Frankfurt, Germany; 2 Department of General and Visceral Surgery, J.W. Goethe-University Hospital, Frankfurt, Germany; 3 Institute of Pathology, J.W. Goethe-University Hospital, Frankfurt, Germany; 4 Institute of Biostatistics and Mathematical Modelling, Faculty of Medicine, J.W. Goethe-University, Frankfurt, Germany; 5 Praxis-Klinik für Diagnostik (PKD) am Staedel, Frankfurt, Germany; Johns Hopkins University, United States of America

## Abstract

**Background:**

Acoustic Radiation Force Impulse (ARFI)-Imaging is an ultrasound-based elastography method enabling quantitative measurement of tissue stiffness. The aim of the present study was to evaluate sensitivity and specificity of ARFI-imaging for differentiation of thyroid nodules and to compare it to the well evaluated qualitative real-time elastography (RTE).

**Methods:**

ARFI-imaging involves the mechanical excitation of tissue using acoustic pulses to generate localized displacements resulting in shear-wave propagation which is tracked using correlation-based methods and recorded in m/s. Inclusion criteria were: nodules ≥5 mm, and cytological/histological assessment. All patients received conventional ultrasound, real-time elastography (RTE) and ARFI-imaging.

**Results:**

One-hundred-fifty-eight nodules in 138 patients were available for analysis. One-hundred-thirty-seven nodules were benign on cytology/histology, and twenty-one nodules were malignant. The median velocity of ARFI-imaging in the healthy thyroid tissue, as well as in benign and malignant thyroid nodules was 1.76 m/s, 1.90 m/s, and 2.69 m/s, respectively. While no significant difference in median velocity was found between healthy thyroid tissue and benign thyroid nodules, a significant difference was found between malignant thyroid nodules on the one hand and healthy thyroid tissue (p = 0.0019) or benign thyroid nodules (p = 0.0039) on the other hand. No significant difference of diagnostic accuracy for the diagnosis of malignant thyroid nodules was found between RTE and ARFI-imaging (0.74 vs. 0.69, p = 0.54). The combination of RTE with ARFI did not improve diagnostic accuracy.

**Conclusions:**

ARFI can be used as an additional tool in the diagnostic work up of thyroid nodules with high negative predictive value and comparable results to RTE.

## Introduction

Thyroid nodules are a common finding in regions with inadequate iodine supply and are reported in 33% of unselected adults between the age of 18–65 years [1]. Ultrasound is an accurate method for the detection of thyroid nodules, but it has a low accuracy for the differentiation between benign and malignant thyroid nodules [2]. Therefore, in patients with normal thyroid stimulating hormone fine-needle-aspiration-biopsy (FNAB) is presently recommended as supplementary diagnostic methods in the evaluation of thyroid nodules with a size of ≥10 mm. In addition, FNAB is advised in nodules smaller than 10 mm with suspicious history or suspicious ultrasound findings [3]–[6]. FNAB is known to have a high specificity (60–98%) but varying sensitivity (54–90%) for the diagnosis of malignant thyroid nodules [7]–[10]. Therefore a relevant number of patients with the final diagnosis of benign thyroid nodules receive thyroid surgery more for diagnostic than for therapeutic purposes.

A classical criterion of malignancy is a hard or firm consistency upon palpation or ultrasound-probe pressure [4], [11]. Previously this attribute was subjective and dependent on the experience of the examiner. However, with the introduction of real-time elastography (RTE) a reproducible qualitative assessment of tissue consistency became available. A meta-analysis of RTE reported a mean sensitivity and specificity for the diagnosis of malignant thyroid nodules of 92%, and 90%, respectively [12]. Nevertheless recently RTE was challenged and criticized for its operator dependency and the evaluation of quantitative elastography instead of qualitative elastography was suggested [13].

Acoustic Radiation Force Impulse (ARFI)-Imaging is a novel ultrasound-based elastography method enabling quantitative measurement of tissue stiffness. Most previous studies evaluated ARFI-imaging for non-invasive assessment of liver fibrosis with promising results. In a previous pilot study [14] the feasibility of ARFI for evaluating the thyroid gland was shown.

The aim of the present study was to evaluate the diagnostic accuracy of ARFI-Imaging for the differentiation of thyroid nodules and to compare it with the well known real-time elastography. While RTE is a qualitative elastography method, ARFI-imaging is a quantitative elastography method.

## Materials and Methods

Informed written consent was obtained from all patients and the study was performed in accordance with the ethical guidelines of the Helsinki Declaration and approved by the local ethics committee of the University of Frankfurt. The protocol for this trial and supporting STARD checklist are available as supporting information (see Checklist S1 and Protocol S1). The study period was from Aug. 2010 to Mar. 2012. All patients presenting to our endocrinology department for work-up of thyroid nodules were evaluated for inclusion in the study. Inclusion criteria were the presence of a thyroid nodule ≥5 mm, normal values of thyroid-stimulating hormone, and FNAB of this nodule performed within the last 6-months or FNAB and/or surgery planned at the time of ultrasound examination and finally performed within the study period. Exclusion criteria were cystic lesions of completely liquid nature, no cytology by FNAB or histology by surgery of the thyroid nodule within the study period, indeterminate cytology by FNAB without repeated FNAB, and suspicious or malignant cytology by FNAB without thyroid operation within the study period.

All patients received an ultrasound of the thyroid gland followed by RTE and ARFI-imaging by 4 examiners with at least 4 years of thyroid ultrasound experience. The examiners were blinded to the results of cytology/histology. Cytology with 6–12 month follow-up ultrasound or histology was used as reference method for the diagnosis of benign thyroid nodules. Histology was used as reference method for the diagnosis of malignant thyroid nodules.

### Fine needle aspiration biopsy (FNAB)/Histology

All included patients received either cytology using FNAB and/or histology from thyroid surgery to verify the diagnosis.

FNAB was performed with a 25-gauge needle attached to a 20 ml-syringe. Adequacy of aspirates was defined according to the guidelines of the Papanicolaou society [15]. Patients with suspicious or malignant cytology were referred to surgery and were only included in the study if surgery was performed within the study period. Patients with nondiagnostic aspirate without repeated FNAB or surgery during the study period were excluded from the study. Cytology and histology was read by experienced pathologists with at least 6 years of working experience who were blinded to the results of ultrasound and elastography.

### Conventional ultrasound (B-mode and Doppler)

All patients received an ultrasound examination of the thyroid gland using a 9-MHz transducer (Hitachi-EUB 900, Hitachi,Tokyo, Japan). The patients were positioned in a supine position with dorsal flexion of the head. Ultrasound was performed by experienced examiners blinded to the results of cytology. Thyroid nodules were evaluated for size, volume, echogenicity, echotexture, presence/absence of halo-sign, presence/absence of microcalcification and/or macrocalcification. After B-mode-ultrasound, power-Doppler and Duplex-imaging were performed [16].

### Real-time Tissue Elastography (RTE)

Real-time elastography (Hitachi Real-time Tissue Elastography [HI-RTE], Hitachi Medical Corporation, Japan) is an imaging technique to directly reveal the physical property of tissue with conventional ultrasound probes. Tissue elasticity distribution is calculated by the strain and stress of the examined tissue. The calculation of tissue elasticity distribution was performed in real-time and the examination results were represented as color-coded images over the conventional B-mode image (blue = hard, red & green = soft tissue). Details have been described in previous studies [17], [18]. Real-time elastography was performed with the EUB-900 ultrasound-system (Hitachi, Tokyo, Japan) using the 9-MHz probe. The probe was placed on the neck and a light pressure of 3–4 on a scale of 0–6 arbitrary units was applied for measurement. The region-of-interest (ROI) for the elastography examination was selected by the operator including the nodule and surrounding normal thyroid tissue. Video clips and single images were digitally stored. Elasticity was classified in four different patterns as described previously [19], [20]: elasticity score (ES)-1: the nodule is displayed homogeneously in green (soft); ES-2: the nodule is displayed predominantly in green with few blue areas/spots; ES-3: the nodule is displayed predominantly in blue with few green areas/spots; ES-4: the nodule is displayed completely in blue (hard). In cases of cystic lesion, the solid component of the nodule was examined to exclude artifacts known to be caused by the cyst. The entire examination lasted approx. 5–10 minutes per patient.

### Acoustic Radiation Force Impulse (ARFI)-Imaging

**Figure 1:**
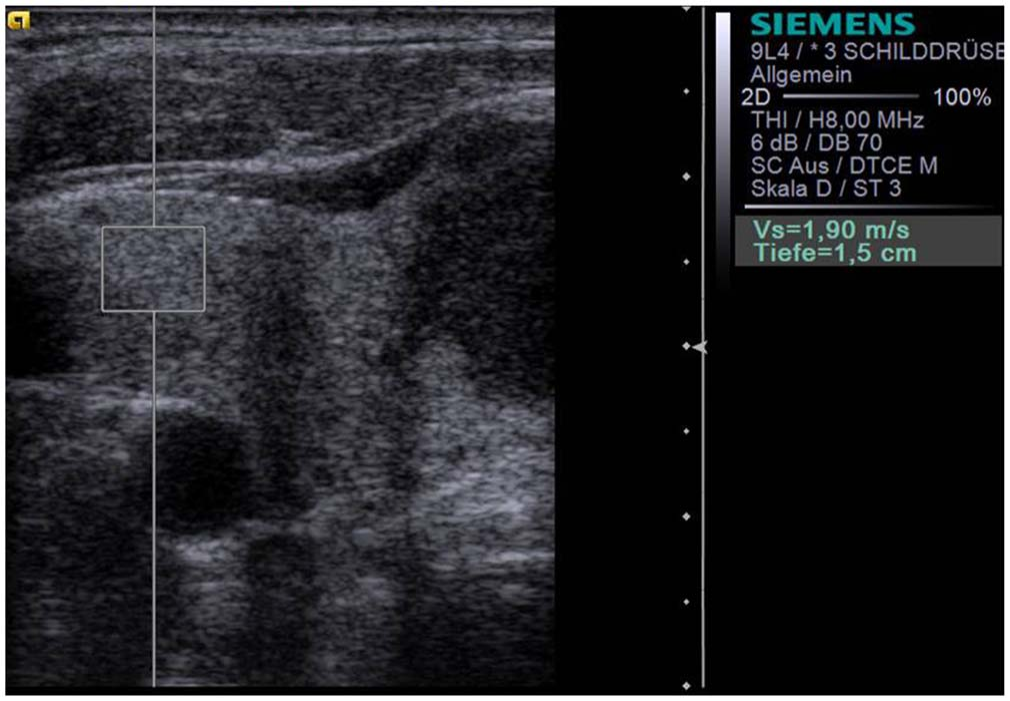
B-mode ultrasound of the right lobe of thyroid gland using the S2000, 9L4 probe at 9 MHz with the ROI placed within the healthy thyroid tissue measuring an ARFI velocity of 1.90 m/s.

ARFI-imaging (Virtual-Touch™-Tissue-Quantification, Siemens-ACUSON-S2000) involves targeting of an anatomic region to be interrogated for elastic properties with a Region-of-Interest(ROI) cursor while performing realtime B-mode-imaging. Tissue at the ROI is mechanically excited using acoustic pulses to generate localized tissue displacements. The displacements result in shear-wave propagation away from the region of excitation and are tracked using ultrasonic, correlation-based methods [21]. The maximum displacement is estimated for many ultrasound tracking beams and the shear wave speed of the tissue can be reconstructed [22], [23]. Using the 9L4-linear ultrasound-probe (5–14 MHz), the shear velocity is estimated within a ROI graphically displayed with a size of 5×5 mm (Figure 1). The shear wave propagation velocity is proportional to the square root of tissue elasticity [24], [25]. Results are expressed in m/s (range: 0.5–8.4 m/s). Value ranges exceeding 8.4 m/s are displayed as “x.xx m/s”.

**Figure 2:**
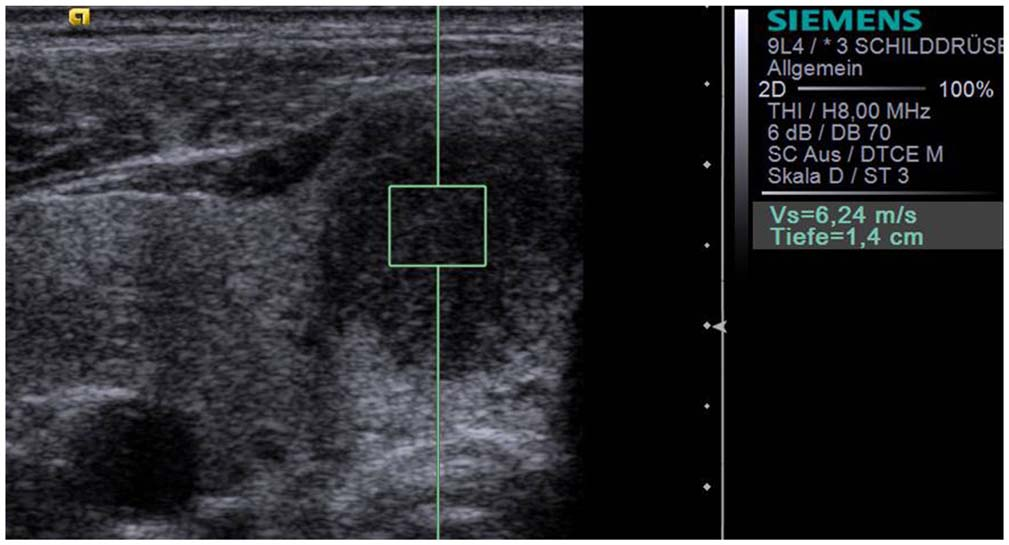
Acoustic Radiation Force Impulse Imaging (S2000, 9L4 probe at 9 MHz) of the same patient a in **Figure 1** **with the ROI placed within a hypoechoic thyroid nodule in the right thyroid lobe measuring a velocity of 6.24 m/s.** Histology revealed papillary carcinoma.

ARFI-imaging was performed with a 9L4-linear-ultrasound-probe at 9-MHz for B-mode imaging. Ten successful measurements were performed with the ROI placed in the healthy thyroid gland away from thyroid nodules (Figure 1). In addition, ten successful measurements were performed with the ROI placed in the thyroid nodule (Figure 2). In cases without 10-valid measurements (numeric value), and more “x.xx m/s” measurements than numeric values, “x.xx m/s” was allocated to be 8.4 m/s to reach 10 numeric values for each nodule.

In addition, the ratio between each nodule and adjacent thyroid tissue was calculated. In patients with goiter without healthy thyroid tissue of 5×5 mm enabling measurement, the median of all healthy thyroid tissue of the entire study population was used for the calculation of ratio.

### Statistical analysis

Statistical analysis was performed using BiAS-for-Windows (version-9.10, epsilon-2011, Frankfurt, Germany). For ARFI-imaging the median of all 10 measurements per subject and nodule or healthy thyroid gland was calculated and used for further analysis. Values of ARFI-imaging were not normally distributed and therefore expressed as median-values. Clinical and laboratory characteristics of patients were expressed as mean ± SD, median and range. Correlations were assessed by Spearman's correlation coefficient. Comparison of patient characteristics was performed using the non-parametric Wilcoxon-Mann-Whitney U-Test (two-sides, level of significance α = 5%) for quantitative values and two-sided Fisher's exact test for qualitative characteristics. A p-value less than 0.05 was judged to be statistically significant.

The diagnostic performance of ARFI-imaging and RTE was also assessed by receiver-operating-characteristic (ROC)-curves. The ROC-curve represents sensitivity versus 1-specificity for all possible cut-off values for prediction of the different fibrosis stages, respectively. Cut-off values for ARFI for the diagnosis of malignant thyroid nodules defined using Youden's-index [26] and sensitivity, specificity, positive and negative predictive values, and positive likelihood ratio (LR) were calculated without further adjustments (e.g. by cross-validation) from the same data. The intra-observer variability was calculated as the mean standard deviation of 10-measurements at one location.

A sample-size-calculation was performed prior to the study performance with the primary endpoint to improve the Youden's-index for the combination of ARFI-imaging and RTE as compared to RTE alone. To improve specificity of the diagnostic methods, the diagnosis of malignancy was assumed if both tests suggested malignancy. Therefore a two-sided comparison of two-binomially distributed values was performed with a level of significance of α = 5%. We assumed 20% of malignant nodules and a reduction of sensitivity by 1% with an increase of specificity for the combination of ARFI and RTE by 20%. Herefore, 135 patients were needed to show improvement of Youden's-index with a statistical-power of 80%.

## Results

One-hundred-fifty-two patients with 173 thyroid nodules were examined according to the study protocol. Study recruitment was from August 2010 to April 2012. Thirteen patients were excluded because of nondiagnostic aspirate on FNAB without repeated FNAB/surgery during the study-period. Two additional patients were excluded because of suspicious or malignant aspirate on FNAB without surgery during the study-period. Therefore, one-hundred-thirty-eight patients with 158 examined nodules were included in the final analysis. Patient characteristics are shown in Table 1. All patients showed up with normal thyroid hormone values.

**Table 1:** Patient characteristics.

Characteristics	All 138 Patients with 158 nodules	122 Patients with 137 benign nodules*	17 Patients with 21 malignant nodules*	p-value
Patient age (years)				0.60
Mean ± SD (range)	52±14,5 (18–83)	52± 14 (18–83)	53± 17 (18–77)	
Median	52	52	56	
Male gender, n (%)	39 (28)	32 (26)	8 (47)	0.090
Single nodule, n (%)	37 (27)	34 (28)	3 (18)	0.41
Goiter, n (%)	101 (73)	88 (72)	14 (82)	
Nodule location				0.15
Left, n (%)	64 (41)	59 (43)	5 (24)	
Right, n (%)	92 (58)	76 (55.5)	16 (76)	
Istmus, n (%)	2 (1)	2 (1.5)	0 (0)	
Nodule size				0.68
Mean ± SD (range)	20±12 (5–85)	20±11 (5–59)	24±21 (5–85)	
Median	17	17	17	
Cytology of nodule, n (%)	124 (78.5)	115 (84)	9 (43)	0.00012
Histology of nodule, n (%)	64 (40.5)	43 (31)	21 (100)	<0.000001
Real-time elastography Score				0.00025
ES 1, n (%)	1 (0.5)	1 (1)	0 (0)	
ES 2, n (%)	103 (65)	98 (71)	5 (24)	
ES 3, n (%)	50 (32)	37 (27)	13 (62)	
ES 4, n (%)	4 (2.5)	1 (1)	3 (14)	
ARFI-Imaging				0.0039
Mean ± SD (range)	2.20±1.31 (0.5–8.4)	2.02±0.95 (0.5–8.4)	3.41±2.37 (0.6–8.4)	
Median	1.91	1.90	2.69	

* *one patient had 1 benign nodule in the left thyroid lobe and two malignant nodules in the right thyroid lobe.*

*SD = standard deviation.*

### Cytology/Histology

FNAB was performed of 124 nodules (114 patients). Hereby, FNAB revealed benign cytology from 107 nodules, suspicious cytology from 14 nodules, papillary carcinoma in 2 nodules and squamous carcinoma cells in 1nodule. All suspicious and malignant nodules on cytology were referred to surgery. Twenty-four additional patients were operated. In 18 of these patients surgery was advised due to goiter with multiple nodules; and in six of these patients FNAB was declined by the patients who preferred direct surgery. Overall 49 patients with 64 examined thyroid nodules were operated. Histology revealed benign adenoma and/or regressive changes in 32 patients with 43 nodules. Papillary carcinoma was found in 12 patients with 13 examined nodules, follicular carcinoma in 2 patients with 4 examined nodules, medullary thyroid carcinoma in 2 patients with 3 examined nodules and anaplastic thyroid carcinoma in one patient with one examined nodule. The time interval between elastography and cytology was 0–6 months and between elastography and operation 0–2 months. No severe complication were observed after FNAB and thyroid surgery.

### Conventional Ultrasound

Results of B-mode ultrasound criteria are shown in Table 2. The highest sum of sensitivity and specificity was found for the combination of the presence of microcalcification and absence of halo sign with 62% sensitivity (95%-CI: 38;82), 80% specificity (95%-CI: 72;86), 93% NPV (95%-CI: 87;97), 32% PPV (95%-CI: 18;48), and 3.03 positive LR (95%-CI: 1.89;4.85), respectively.

**Table 2:** Senstivity, specificity, PPV and NPV for thyroid cancer for different ultrasound patterns (including RTE and ARFI) in thyroid nodules.

criteria	Benign (n = 137)	Cancer (n = 21)	Sens (%)	Spec (%)	NPV (%)	PPV (%)	+LR
Hypoechogenicity							
Yes	48	13	62	65	92	21	1.77
No	89	8	(38;82)	(56;73)	(84;96)	(12;34)	(1.18;2.65)
Microcalcifications							
Yes	45	15	71	67	94	25	2.18
No	92	6	(48;89)	(58;75)	(87;98)	(15;38)	(1.52;3,12)
Absent Halo sign							
Yes	86	19	90	37	96	18	1.44
No	51	2	(70;99)	(29;46)	(87;99.5)	(11;27)	(1.19;1.74)
Irregular margins							
Yes	26	11	52	81	92	30	2.76
No	111	10	(30;74)	(73;87)	(85;96)	(16;47)	(1.62;4.71)
Oval shape							
Yes	75	11	52	45	86	13	0.96
No	62	10	(29;74)	(37;54)	(76;93)	(7;22)	(0.62;1.48)
Pattern 3–4 vasc.							
Yes	37	11	52	73	91	23	1.94
No	100	10	(30;74)	(65;80)	(84;96)	(12;37)	(1.18;3.17)
ES 3–4							
Yes	38	16	76	72	95	30	2.75
No	99	5	(53;92)	(64;80)	(89;98)	(18;44)	(1.92;3.94)
ARFI ≥2.57 m/s							
Yes	20	12	57	85	93	38	3.91
No	117	9	(34;78)	(78;91)	(87;97)	(21;56)	(2.26;6.78)
ARFI ratio ≥1.57							
Yes	22	12	57	84	93	35	3.56
No	115	9	(34;78)	(77;90)	(87;97)	(20;54)	(2.01;6.06)
ES 3–4+ARFI ≥2.57 m/s							
Yes	11	10	48	92	92	48	5.93
No	126	11	(26;70)	(86;96)	(86;96)	(26;70)	(2.88;12.22)
E S 3–4+ARFI ≥1.11 m/s							
Yes	34	16	76	75	95	32	3.07
No	103	5	(53;92)	(67;82)	(90;99)	(20;47)	(2.11;4.48)

*ARFI = Acoustic Radiation Force Impulse Imaging; ES = real-time elastography score; LR = likelihood ratio; US = ultrasound; Sens = sensitivity; Spec = specificity, PPV = positive predictive value; NPV = negative predictive value; vasc. = vascularisation.*

### Real-time Elastography (RTE)

RTE score ES-1 was found in one benign nodule; ES-2 in 103 nodules (98 benign nodules; 2 papillary carcinoma, 2 follicular carcinoma and one medullary carcinoma); ES-3 in 50 nodules (37 benign nodules, 9 papillary carcinoma, 2 medullary carcinoma, one follicular carcinoma, and one anaplastic carcinoma); and ES-4 in one benign nodule, 2 papillary carcinoma, and one follicular carcinoma, respectively.

Thus, 99/158 nodules (63%) with the final diagnosis of benign nodules showed ES-1&2, and 16/21 nodules (76%) with the final diagnosis of thyroid cancer showed ES-3&4.

Using ES-3&4 for the diagnosis of malignant thyroid nodules and ES-1&2 for the diagnosis of benign thyroid nodules, NPV of RTE was 95%. Details are shown in Table 2.

The highest sum of sensitivity and specificity was found for the combination of ES3–4 with the absence of halo-sign with 71% sensitivity (95%-CI: 48;89), 81% specificity (95%-CI: 73;87), 95% NPV (95%-CI: 89;98), 37% PPV(95%-CI: 22;53), and 3.76 positive LR(95%-CI: 2.43;5.84), respectively.

### Acoustic Radiation Force Impulse (ARFI)-Imaging

Velocities of ARFI measured in thyroid nodules and tissue are shown in Table 3. In 5 patients no measurement in the healthy thyroid gland was possible due to multinodular goiter. While no significant difference in median velocity was found between healthy thyroid tissue and benign thyroid nodules (p = 0.068), a significant difference was found between healthy thyroid tissue and malignant thyroid nodules (p = 0.0019), as well as between benign and malignant thyroid nodules (p = 0.0039), respectively.

**Table 3:** ARFI velocity characteristics.

ARFI velocity (m/s)	Healthy thyroid	Benign nodule	Malignant nodule
Mean ± Standard deviation	1.80±0.42	2.02±0.95	3.41±2.37
Median	1.76	1.90	2.69
Minimum	0.89	0.50	0.61
Maximum	3.33	8.40	8.40

*p-value: p = 0.068 between healthy thyroid vs. benign nodule; p = 0.0019 between healthy thyroid vs. malignant nodule; p = 0.0039 between benign and malignant nodules.*

The median success-rate of ARFI-measurement (number of valid measurements divided by the number of all measurements performed) was 100% in the healthy thyroid (mean: 99±2%, range: 91–100%), and 100% in thyroid nodules (mean: 92±17%,range: 9–100%). The lower mean success-rate for nodules accounts for the upper measurement limit of 8.4 m/s above which values were displayed as “x.xxm/s” and therefore counted as unsuccessful measurement.

**Figure 3:**
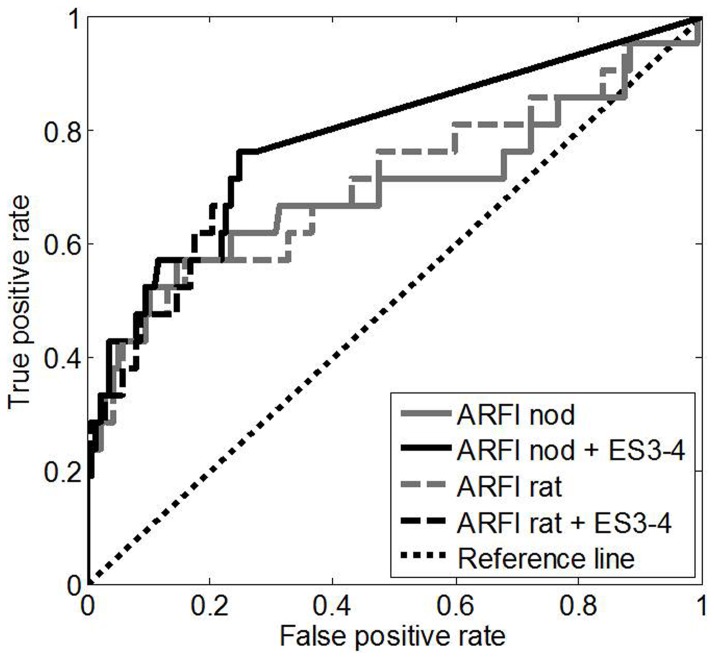
Receiver-operating characteristic (ROC) curves for ARFI-imaging of thyroid nodule (ARFI-nod) and for the ratio of ARFI-imaging of thyroid nodule divided by ARFI-imaging of healthy thyroid tissue (ARFI-rat) for diagnosis of malignant thyroid nodules (AUROC 69% and 71%, respectively) and the ROC curves for the combination of these criteria with Realtime Elastography Score (ES) 3–4 (AUROC 78% and 78%, respectively). The improvements with respect to ARFI alone were not significant (p = 0.18 for ARFI-nod, p>0.20 for ARFI-rat), analogously the improvements with respect to ES alone were not significant (p>0.20 in both cases).

AUROC for ARFI of the thyroid nodule for the diagnosis of malignant thyroid nodules was 0.69 [95-CI: 0.53;0.85] (p = 0.0043). The optimal cut-off with the highest sum of sensitivity and specificity (Youden cut-off) for ARFI-measurement in thyroid nodules was 2.57 m/s (Table 2). AUROC for the ratio of ARFI in the nodule and healthy thyroid tissue for the diagnosis of malignant thyroid nodules was 0.71 [95-CI: 0.56;0.85] (p = 0.0025). The optimal cut-off (Youden cut-off) for ARFI-ratio was 1.57 m/s (Table 2). No significant difference was found between AUROC of ARFI of the nodule and ARFI-ratio (p>0.20). Details are shown in Table 2 and Figure 3.

Intra-observer variability expressed as the mean standard deviation of 10 measurements at one location was 0.46 within all thyroid nodules, and 0.21 within healthy thyroid tissue. It was higher in malignant nodules with 0.94 as compared to benign nodules with 0.39.

### Comparison and Combination of RTE and ARFI-Imaging

Spearman correlation coefficient between the velocity measured with ARFI-imaging and the elasticity score measured with RTE was significant with 0.30 (p = 0.00016).

AUROC for the diagnosis of malignant thyroid nodules of RTE was 0.74 [95%-CI: 0.64;0.84] (p = 0.00038). No significant difference in AUROC was found between ARFI and RTE (0.69 vs. 0.74, p>0.20).

When combining ARFI and RTE (ES 3–4 and ARFI ≥2.57 m/s- both must be present) specificity for the diagnosis of malignant thyroid nodules increased by 20% as compared to RTE alone (92% vs. 72%). However, sensitivity decreased by 28% (48% vs.76%) and the Youden index did not improve (p>0.20). When optimizing the cut-off of ARFI for the combined decision (ES 3–4 and ARFI ≥1.11 m/s-both must be present) sensitivity remained 76% but specificity only increased by 3% as compared to RTE alone (75% vs. 72%) and again the Youden's-index did not improve (p>0.20, s.Tab.2). No significant improvement of diagnostic accuracy was found for the combination of ARFI and RTE as compared to both methods alone (78% vs. 69%, p = 0.18, 78% vs. 74%, p>0.20).

## Discussion

RTE has become a well evaluated clinical tool enabling the determination of tissue elasticity using ultrasound devices. RTE is a qualitative elastography method evaluating changes in ultrasound pattern during strain and stress of direct or indirect tissue compression. A recent meta-analysis reported a sensitivity and specificity for RTE for the diagnosis of malignant thyroid nodules of 92%, and 90%, respectively [12]. Methods to quantify the colour coded images revealed by RTE were developed using strain value and ratio and histograms with the aim of reducing intra- and interobserver variability [27]–[29]. Nevertheless, besides a lot of promising study results two recent studies have challenged the usefulness of RTE in clinical practice by reporting no additional value as compared to qualified B-mode ultrasound [13], [30].

Quantitative elastography was well evaluated for the diagnosis of liver fibrosis with most studies evaluating transient elastography (FibroScan, Echosens, Paris) [31]. Hereby, a mechanical wave is send into the liver and the velocity of shear waves within the liver is measured. However, it was only developed for measurement in liver tissue. Recently, other quantitative elastography methods were developed, which are integrated in conventional ultrasound systems and can be performed in all solid organs [32], [33]. These quantitative methods also send a mechanical or acoustic wave into the tissue and measure the velocity of shear waves; the stiffer the tissue is, the faster the shear waves propagate. Only one previous study with 146 nodules from 93 patients evaluated shear wave elastography with SuperSonic Imaging (Aixplorer, Aixen Provence, France) and reported a sensitivity of 85%, and a specificity of 94% for the diagnosis of malignant thyroid nodules [34]. In a recently published pilot study [14] the feasibility of Acoustic Radiation Force Impulse (ARFI)-imaging to measure thyroid tissue and thyroid nodules was shown. To our knowledge, no previous study has compared the well evaluated qualitative RTE and the novel quantitative elastography methods for the differentiation of thyroid nodules. The results of the present study show comparable results for RTE and ARFI-imaging for the differentiation of benign and malignant thyroid nodules. Sensitivity and specificity of RTE in the present study was lower than in the published meta-analysis on RTE [12] with sensitivity of 76% vs. 92%, and specificity of 72% vs. 90%. However, the results or RTE in the present study were higher than in the study of Moon et al. [30] evaluating 703 nodules in 676 patients with sensitivity of 75% vs. 65%, and specificity of 72% vs. 58%. The results and these discrepancies again might be explained by the qualitative and operator-depending procedure of RTE. The advantage of ARFI-imaging is that the same acoustic wave is send into the tissue independent of the examiner pressing the button to start measurement, while for RTE the examiner needs to perform small compressions to the tissue which may vary. In addition, RTE determines tissues elasticity in relation to surrounding tissue, whereas ARFI is a quantitative method measuring the velocity of shear waves within a ROI.

The combination of RTE with ARFI-imaging improved specificity for the diagnosis of malignant thyroid nodules from 72% (RTE alone) to 92% (combination of both), but reduced sensitivity from 76% to 48%, respectively. Both methods revealed an excellent negative predictive value for excluding malignant thyroid nodules with 95% for RTE alone, and 93% for ARFI alone. The combination of both methods did not further improve NPV.

A possible clinical algorithm could be to use primarily one elastography method in combination with FNAB to exclude malignancy of a thyroid nodule and perform follow-up examinations in patients with benign FNAB and benign criteria on RTE or ARFI. However, both methods might be useful in combination if FNAB reveals benign cytology, but one elastography method shows criteria of malignancy. If then both methods (RTE and ARFI) report values in the range of malignancy, than operation could be advised despite the benign cytology. Nevertheless, of course B-mode ultrasound criteria must be included in such an algorithm. Further larger studies are necessary to find an optimal algorithm of B-mode ultrasound, qualitative and quantitative elastography and FNAB to optimize the work up of thyroid nodules.

The present study has the following limitations:

The reference standard was cytology only in 94/158 (59.5%) nodules with benign cytology. However ultrasound examination after 6 months did not show growth of nodule size as a sign of benign lesions. Nevertheless, false-negative cytology may have existed. Histology was the only excepted reference method for the diagnosis of malignant thyroid nodules. The malignant nodules were predominantly papillary carcinoma which might limit the diagnostic utility to this entity.

Cystic lesions without at least 5×5 mm of solid parts of the nodules were excluded from the present study, since ARFI –ROI measures 5×5 mm and cystic lesions produce artefacts on RTE mimicking hard tissue. Therefore, the results of the present study cannot draw any conclusion concerning the value of ARFI for predominantly cystic lesions.

The guidelines for clinical practice for the diagnosis and management of thyroid nodules of the American Association of Clinical Endocrinologists (AACE), Associazione Medici Endocrinologi (AME) and the European Thyroid Association recommend that suspicious thyroid nodules smaller than 10 mm should be assessed by FNAB [5]. However, in the present study, only 22 nodules with 5–10 mm in size were included, which was too small to perform a subanalysis. A recent study demonstrated, that RTE can be performed in thyroid nodules of 3–10 mm in size and is suitable for the diagnosis of microcarcinoma of the thyroid gland [35]. Future studies should evaluate the value of ARFI and the combination of ARFI with RTE in thyroid nodules <10 mm.

The intra-observer variability expressed as the mean standard deviation of 10 measurements at one location was 0.46 within thyroid nodules, and 0.21 within healthy thyroid tissue. Especially in malignant thyroid nodules it was as high as 0.94. A reason might be that many measurements in malignant nodules resulted in “x.xx m/s” if the value exceeded the upper detection limit of 8.4 m/s. In these cases more than 10 measurement attempts were made to reach 10 numeric values. A software optimization increasing the velocity detection at velocities exceeding 8.4 m/s are needed to overcome this limitation.

In summary, the present study demonstrates comparable results for the novel quantitative Acoustic Radiation Force Impulse-Imaging as for the well evaluated qualitative real-time elastography for the differentiation of thyroid nodules. The combination of both methods did not significantly improve the diagnostic accuracy for the diagnosis of malignant thyroid nodules. Large multicenter studies are necessary to develop application algorithms for qualitative and quantitative elastography in clinical practice.

## Supporting Information

Checklist S1 **STARD Checklist.**
(DOC)

Protocol S1 **Trial Protocol.**
(PDF)
