# Supplementary material for: Acoustic Radiation Force Impulse Imaging for Differentiation of Thyroid Nodules
Source: PLoS One. 2012 Aug 29;7(8):e42735. doi: 10.1371/journal.pone.0042735 (PMC3430659; doi:10.1371/journal.pone.0042735)
Supplement: Protocol S1 — Trial Protocol. (PDF) [file pone.0042735.s002.pdf]

Studienprotokoll

Version 2.0 vom 23.11.2009

„Realtime-Elastographie und Acoustic Radiation Force Impulse (ARFI)-Elastographie für die Differenzierung von Schilddrüsen-Knoten“

Leiter der Studie:

Priv.-Doz. Dr. med. Jörg Bojunga

Oberarzt der Medizinische Klinik 1

Klinikum der J.W. Goethe-Universität

Theodor-Stern-Kai 7

60590 Frankfurt am Main

Tel: +49-69-6301-87681

Fax: +49-69-6301-6448

Vertraulich

Diese Unterlagen sind Eigentum der Medizinischen Klinik 1 der Johann Wolfgang Goethe Universität Frankfurt. Alle Informationen sind vertraulich und dürfen nur in Belangen der Studie verwendet werden. Sie dürfen ohne schriftliche Genehmigung der Studienverantwortlichen nicht an Dritte weitergegeben werden.

Prüfplan

Studientitel:

„Realtime-Elastographie und Acoustic Radiation Force Impulse (ARFI)-Elastographie für die Differenzierung von Schilddrüsen-Knoten“

1. Prüfer

Leiter der Studie:

Priv.-Doz. Dr. med. Jörg Bojunga, Oberarzt der Medizinischen Klinik 1, Johann Wolfgang Goethe-Universität Frankfurt

Prüfärzte an der Medizinischen Klinik 1:

Dr. Mireen Friedrich-Rust, Dr. Gesine Meyer, Dr. Sonja Weber, Dr. Andreas Polta

Verantwortliche Biostatistikerin:

Prof. Dr. Eva Herrmann, Institut für Biostatistik und Mathematische Modellierung, Fachbereich Medizin, J.W.Goethe-Universität

Version vom 2.0 vom 23.11.2009

Hiermit versichere ich, dass das hier vorgelegte Studienprotokoll von mir gelesen und in seiner endgültigen Fassung zur Einreichung bei der lokalen Ethikkommission bestimmt ist.

Ort, Datum	Priv.-Doz. Dr. Jörg Bojunga<br>Verantwortlicher Studienleiter

Ort, Datum	Prof. Dr. Eva Herrmann<br>Verantwortliche Biometrikerin

Ort, Datum	Prof. Dr. Stefan Zeuzem<br>Direktor der Klinik

2. Hintergrund

Der sonographische Nachweis nodöser Schilddrüsenveränderungen ist eine der häufigsten Diagnosen in Gebieten mit inadäquater Jodversorgung. Epidemiologische Studien in Deutschland haben nachgewiesen, dass mittels hochauflösender Sonographie bei unselektionierten Probanden im Alter von 18-65 Jahren durchschnittlich in ca. 33 % Schilddrüsenknoten nachweisbar sind (1) mit einem deutlich höherem Risiko für Frauen und ältere Menschen. Der hohen Sensitivität der Sonographie für den Nachweis von Knoten der Schilddrüse steht die geringe Spezifität bezüglich der Zuschreibung ihrer Dignität gegenüber (2). Als ergänzende diagnostische Verfahren werden daher die Szintigraphie sowie die Feinnadelaspirationszytologie (FNAB) eingesetzt.

Nach Leitlinie wird eine Szintigraphie bei sonographischem Nachweis von Schilddrüsenknoten ≥ 10mm empfohlen (3). Szintigraphisch hyperfunktionelle Knoten bergen im Gegensatz zu hypofunktionellen Knoten nur ein sehr geringes Malignitätsrisiko. Demgegenüber kann bei normofunktionellen und hypofunktionellen Knoten nicht automatisch von einem Normalbefund bzw. erhöhten Malignomrisiko ausgegangen werden, da auch die Szintigraphie mit einem prädiktiven Wert zur Malignomdetektion < 10% keine sichere Dignitätsbeurteilung erlaubt (4-6).

Prinzipiell stellt daher jeder Schilddrüsenknoten > 1cm, der szintigraphisch nicht hyperfunktionell ist, eine Indikation zur Feinnadelaspirationszytologie (FNAB) als bisher spezifischste (72-100%), jedoch nicht befriedigend sensitive (65-98%) diagnostische Methode dar (7). Studien haben nachgewiesen, dass sich in 64% der punktierten Knoten eine benigne Zytologie zeigt, 11% sind als suspekt einzustufen und 4% ergeben einen malignen Befund. Für die 21% nicht verwertbaren Ergebnisse wird die Re-Biopsie empfohlen (8;9).

Ziel der Studie ist die Evaluation neuer sonographischer Verfahren der Realtime Elastographie und der Acoustic Radiation Force Impulse (ARFI)-Imaging in der Differentialdiagnose von Schilddrüsenknoten durch Korrelation mit den aspirationszytologischen und histologischen Befunden.

B-Mode Sonographie

Die hochauflösende B-Mode Sonographie ist die sensitivste Methode zum Nachweis von Schilddrüsenknoten (10). Der hohen Sensitivität der Methode steht die weiterhin nicht befriedigende Spezifität in der Differenzierung benigner und maligner Knoten gegenüber (11;12). Ein typisches sonographisches Malignitätszeichen ist die unscharfe und unregelmäßige Knotenbegrenzung sowie organüberschreitendes Wachstum (13). Diese Kriterien sind jedoch häufig auch bei malignen Knoten im Rahmen der Erstdiagnose nicht nachweisbar.

Als sonographische Malignitätshinweise in der B-Mode Sonographie gelten die Knotenmorphometrie mit einem anteroposterior-transversal Durchmesserquotienten > 1 (14;15), Mikrokalzifikationen sowie eine verminderte Echogenität (16;17).

In der vorliegenden Studie werden in der B-Mode Sonographie neben morphometrischen Kriterien die Knotenbegrenzung, Echogenität, Vorhandensein von Mikroverkalkungen sowie das Verhältnis von anteroposteriorem zu transversalem Durchmesser erhoben.

Power-doppler Sonographie

Neben der konventionellen B-Mode Sonographie stellt die farbkodierte Doppler-Sonographie bzw. power-Doppler Sonographie eine wichtige ergänzende Untersuchung zur Evaluation der Schilddrüsen- und Knotenperfusion dar. Durch zusätzliche Anwendung dieser Methoden kann die Sensitivität und Spezifität der Sonographie in der Detektion maligner Läsionen verbessert werden (18).

Das typische doppler-sonographische Kriterium der Benignität von Knoten ist die ausschließlich perinoduläre Vaskularisation, wohingegen der Nachweis einer intranodalen Vaskularisation mit einer odds-ratio (OR) von 14.2 ein evaluiertes sonographisches Malignitätskriterium darstellt (19). Auch das Perfusionsmuster und die Perfusionsintensität - gemessen anhand semiquantitativer Skalen - können eine Verbesserung der Spezifität im Nachweis von Malignomen erbringen (20), obwohl andere Autoren in bis zu 14% über eine fehlende Mehrperfusion maligner Knoten in der Doppler-Sonographie berichten (21). Neuere Arbeiten haben daher den Stellenwert velocimetrischer Parameter in der Prädiktion maligner Knoten evaluiert

und nach Bildung von cut-off Werten über eine verbesserte diagnostische Spezifität des Ultraschalls unter Verwendung der pw-Doppler Parameter des Resistance- und Pulsatilitäts-Index berichtet (22;23).

In der vorliegenden Studie werden mittels power-doppler Sonographie Perfusionsmuster (perinodal, intranodal), Perfusionsintensität anhand einer semiquantitativen Skala erhoben.

Realtime Elastographie

Die Palpation ist die älteste Methode zum Nachweis von Schilddrüsenknoten. Ein charakteristisches Kriterium maligner Schilddrüsenknoten ist dabei die im Vergleich zu benignen Knoten deutlich härtere Konsistenz (24). Die Darstellung der Gewebeelastizität würde daher einen potenziell wichtigen Beitrag für die Differentialdiagnostik von Schilddrüsenknoten liefern. Sonographische Verfahren zur Abbildung der elastischen Gewebeeigenschaften wurden in den letzten Jahren entwickelt und experimentell evaluiert. Das Elastizitätsmodul, d.h. die Gewebeelastizität, läßt sich aus den Dehnungs- und Belastungswerten der untersuchten Gewebestrukturen ableiten. Neue Verfahren ermitteln die Elastizitätswerte der untersuchten Gewebestruktur über eine sog. erweiterte kombinierte Autokorrelationsmethode in Verbindung mit einer 3D-Finite-Elemente-Berechnung (25). Dies ermöglicht eine genaue Abschätzung der Belastungswerte, und der Einfluss der seitlichen Verschiebung kann zufriedenstellend kompensiert werden. Die Untersuchung ist einfach mit Standardschallsonden ohne zusätzliche Apparaturen (Systeme zur Messung der eingeleiteten Drucke, Vibrationsquellen etc.) durchzuführen, ähnlich einer Farbdoppleruntersuchung. Die Berechnung der Elastizitätswerte erfolgt in Echtzeit, die Ergebnisse werden dem konventionellen B-Bild farbkodiert überlagert.

Aufgrund der biologischen Eigenschaften maligner Schilddrüsenknoten erscheint der Einsatz der Realtime Elastographie in der Differentialdiagnose von Schilddrüsenknoten viel versprechend. Erste Studien unter Einsatz der Realtime Elastographie berichten über Sensitivitäten von 82-97% und Spezifitäten von 78-100%, jedoch den meisten um 80% für die Diagnose eines Schilddrüsenkarzinoms (26-30).

In der vorliegenden Studie wird der Stellenwert der neuen ARFI-Imaging im Vergleich zur Realtime Elastographie in der Differenzierung von Schilddrüsenknoten evaluiert.

Acoustic Radiation Force Impulse (ARFI)-Imaging

Acoustic Radiation Force Impulse Imaging (ARFI) ist eine neue Technologie (Siemens ACUSON S2000 Virtual TouchTM Tissue Quantification), welche in ein Ultraschallgerät integriert ist und mit konventionellen Ultraschallköpfen durchgeführt werden kann. Die Methode ist CE-zertifiziert. Bei der Acoustic Radiation Force Impulse (ARFI)- Imaging werden akustische Kurzimpulse ins untersuchte Gewebe ausgesandt, welche lokalisierte mikrometer-große Gewebeverschiebungen induzieren. Diese Verschiebungen führen zur Ausbreitung von Transversalwellen weg von der Region der Erregung. Die Ausbreitungsgeschwindigkeit dieser Transversalwellen wiederum wird mittels Ultraschallwellen detektiert und in m/s gemessen. Je steifer das Gewebe desto schneller ist die Ausbreitungsgeschwindigkeit. Es sollten 10 valide Messungen pro Patient durchgeführt werden. ARFI-imaging ist wie die Realtime-Elastographie in ein konventionelles Ultraschallsystem integriert.

In einer ersten Pilotstudie unserer eigenen Arbeitsgruppe konnten wir eine signifikante Korrelation der ARFI-Imaging mit dem histologischen Fibrosestadium bei 85 Patienten mit viraler Hepatitis aufzeigen. Die diagnostische Genauigkeit gemessen mit der Fläche unter der ROC-Kurve (AUROC) betrug 82% für die Diagnose einer signifikanten Fibrose und 91% für die Diagnose einer Leberzirrhose (31). Studien zur ARFI-Imaging bei Schilddrüsenknoten liegen nicht vor.

In der vorliegenden Studie wird der Stellenwert der neuen ARFI-Imaging im Vergleich zur Realtime Elastographie in der Differenzierung von Schilddrüsenknoten evaluiert.

3. Hypothese

Basierend auf diesen Vorarbeiten ist nun das Ziel der vorliegenden Studie in einem Patientenkollektiv mit Schilddrüsenknoten von mindestens 1cm Durchmesser die Ultraschall-basierten Verfahren Realtime-Elastographie und ARFI-Imaging durchzuführen und zu vergleichen unter Verwendung der aspirationszytologischen und histologischen Befunde als Referenzmethode.

4. Zielgrößen

Primäre Endpunkte:

- Bestimmung von Sensitivtät plus Spezifität bzw. des Youden-Index auf Basis der Realtime-Elastographie sowie auf Basis einer Kombination von Acoustic Radiation Force Impulse (ARFI)- Imaging in Ergänzung zur Realtime-Elastographie zur Differenzierung von Schilddrüsenknoten unter Verwendung der aspirationszytologischen und histologischen Befunde als Referenzmethode. Prüfziel ist der Nachweis eines verbesserten Youden-Index für die kombinierte Untersuchung.

Sekundäre Endpunkte:

- Weitere diagnostische Kenngrößen (Sensitivität, Spezifität, positiver prädiktiver Wert, negativer prädiktiver Wert) für ARFI-Imaging und Realtime-Elastographie alleine und in Kombination für die diagnostische Genauigkeit in der Differenzierung von Schilddrüsenknoten

5. Untersuchungsdesign

Die Studie ist als offene, prospektive, monozentrische Studie geplant.

Eingeschlossen werden sollten insgesamt 135 Patienten mit Schilddrüsenknoten von ≥ 1cm, bei denen eine aspirationszytologische Diagnostik erfolgt oder geplant ist oder bei denen eine Schilddrüsenoperation geplant ist. Der Abstand der nicht-invasiven Untersuchungen von der aspirationszytologische Diagnostik darf 6 Monate nicht überschreiten.

Alle Patienten erhalten eine B-Bild Sonographie, eine power-doppler Sonographie, eine Realtime Elastographie und eine ARFI-Imaging von mindestens einem sonographisch auffälligen Knoten (s. diagnostisches Ablaufschema).

In Anlehnung an die Leitlinien der Deutschen Gesellschaft für Endokrinologie wird die Diagnose eines malignen Schilddrüsenknotens nur an Hand der Histologie nach Schilddrüsen-Operation gestellt. Die Diagnose eines benignen Schilddrüsenknotens wird gestellt, wenn entweder der Knoten operiert wurde und die Histologie benigne ist oder die Feinnadelaspirationszytologie eine benigne Zytologie ohne Anhalt auf

Malignität ergibt und der Schilddrüsenknoten nach 6 Monaten sonographisch kein Größenwachstum zeigt.

Diagnostisches Ablaufschema in Anlehnung an die Leitlinien der Deutschen Gesellschaft für Endokrinologie.

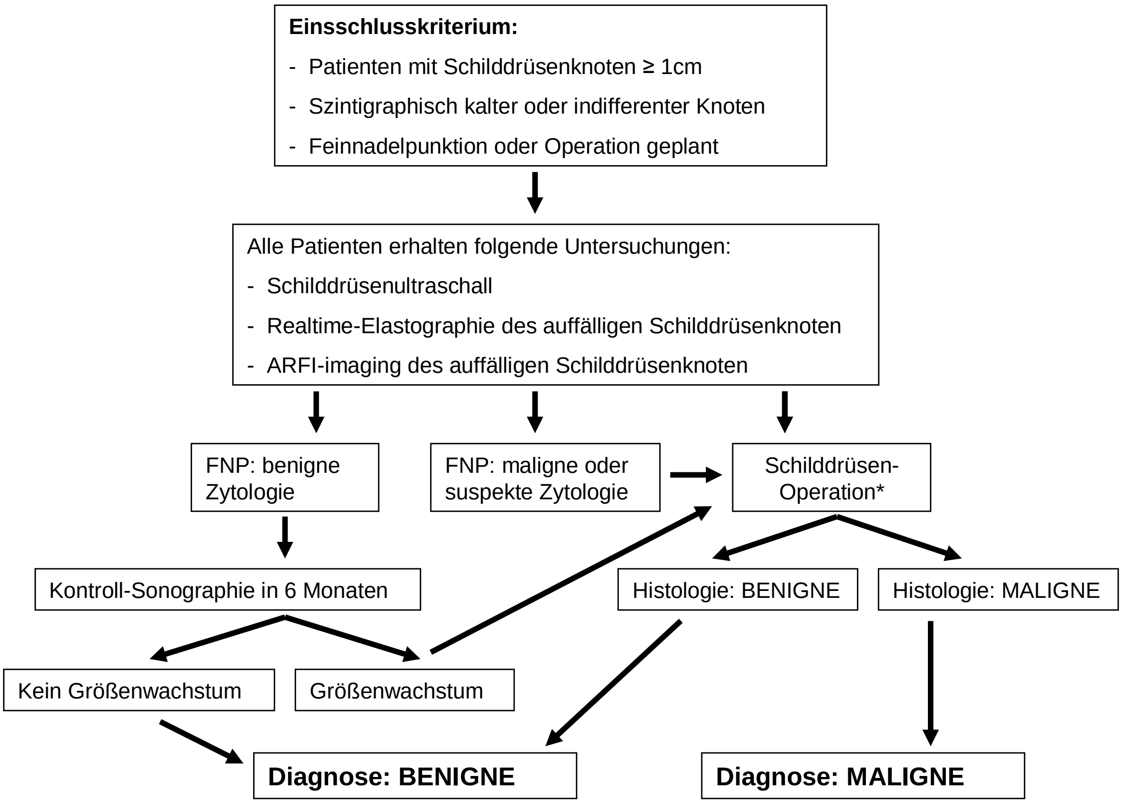

**Indikation zur Schilddrüsen-Operation unabhängig von den Ergebnissen der Realtime-Elastographie oder ARFI (z.B. Struma nodosa, FNP suspekt oder maligne, Patientenwunsch*

6. Statistik und Fallzahlberechnung

Haupt-Prüfziel ist der Nachweis eines verbesserten Youden-Index für die kombinierte Untersuchung mit ARFI-Imaging und Realtime-Elastographie im Vergleich zur Untersuchung mit Realtime-Elastographie alleine.

Um insbesondere die Spezifität der Diagnoseverfahren zu verbessern, wird eine Entscheidung auf der Basis der kombinierten Ergebnisse darin bestehen, nur dann

von malignen Knoten auszugehen, wenn sowohl ARFI-Imaging als auch Realtime-Elastographie für einen malignen Knoten sprechen.

Damit entspricht ein Vergleich des Youden-Index für die Kombination im Vergleich zur Entscheidung auf der Basis der Realtime-Elastographie alleine dem Vergleich zweier binomialverteilter Größen mit einem Standardtest. Für diesen Test wird ein Signfikanzniveau von α=5% angenommen und er soll zweiseitig durchgeführt werden.

Für die Fallzahlberechnung setzen wir voraus, dass der Anteil der malignen Knoten bei 20% liegt und der Verlust in der Sensitivität bei nur 1% aber der Gewinn in der Spezifität für das kombinierte Verfahren um 20% höher ist. Dann werden insgesamt 135 Patienten benötigt, um mit einer Power von 80% eine Verbesserung des Youden-Index statistisch nachzuweisen.

Die statistische Auswertung wird unter Betreuung und Beratung von Mitgliedern des Instituts für Biostatistik und mathematische Modellierung des Fachbereichs Medizin der Goethe-Universität durchgeführt.

7. Anzahl der Patienten

In die Studie sollen insgesamt 135 Patienten mit Schilddrüsenknoten, die sich in unserer endokrinologischen Ambulanz oder Station vorstellen, prospektiv eingeschlossen werden.

8. Zeitrahmen

Für die Rekrutierung ist ein Zeitraum von zwei Jahren vorgesehen. Beginn der Rekrutierung ist der 01.12.2009.

9. Ein- und Ausschlusskriterien

Einschlusskriterien sind:

1. Patienten mit Schilddrüsenknoten ≥ 1cm

2. aspirationszytologische Diagnostik erfolgt oder geplant oder Schilddrüsenoperation geplant
3. Alter 18-79 Jahre
4. unterzeichnete Einverständniserklärung

Ausschlusskriterien sind:

1. Erkrankungen, die die Studiendurchführung unmöglich machen oder für den Patienten ein Risiko darstellen (z.B. psychiatrische Erkrankungen)
2. nicht geschäftsfähige Patienten
3. Schwangerschaft
4. Stillzeit
5. Patienten ohne aspirationszytologische oder histologische Befunde
6. Szintigraphisch heiße Knoten

10. Erfassen der Zielgrößen

Sonographie eines Schilddrüse

- Seite de untersuchten Knotens
- Struma oder solitärer Knoten
- Schilddrüsenvolumen
- Echogenität der Schilddrüse
- Größe des Knotens (Länge, Breite, Tiefe) in cm
- Vorhandensein eine Halosaums
- Begrenzung des Knotens
- Form des Knotens
- Echgenität des Knotens
- Homogenität des Knotens
- Vorhandensein von Makro- oder Mikroverkalkungen im Knoten
- Zystische Veränderungen im Knoten
- Power-Doppler-Perfusion des Knotens

Realtime-Elastographie

- Angabe eines Elastographie-Scores (ES):
  - ES 1: elastographisch homogen grüner knoten (weich)
  - ES 2: elastographisch vorwiegend grüner Knoten mit blauen Arealen oder Punkten
  - ES 3: elastographisch vorwiegend blauer Knoten mit einzelnen grünen Arealen oder Punkten
  - ES 4: homogen blauer Knoten

ARFI-Imaging Knoten

- Ergebnisse der 10 erfolgreichen Einzelmessungen in m/s
- Zahl der Fehlmessungen markiert durch XXXX.

ARFI-Imaging gesunde Schilddrüse

- Ergebnisse der 10 erfolgreichen Einzelmessungen in m/s
- Zahl der Fehlmessungen markiert durch XXXX.

Anamnestische Angaben und körperliche Untersuchungsbefunde:

Folgende anamnestischen Angaben und Untersuchungsbefunde werden am Tag der ARFI-Imaging und transienten Elastographie erhoben:

- Datum der Erstdiagnose des Schilddrüsenknotens
- Nachweis eines Wachstum des Knotens
- Vorhandensein eines Rezidiv-Knotens nach OP
- Vorangegangene Radiatio
- Einnahme von Schilddrüsenmedikamenten
- Gewichtsverlust
- Karzinom/Metastase in der Vergangenheit

11. Besondere Risiken

Es sind bisher keine Nebenwirkungen der Realtime-Elastographie oder der Acoustic Radiation Force Impulse Imaging-Untersuchung berichtet worden. Beide Geräte sind CE-zertifiziert. Da bislang noch keine Untersuchungen bei schwangeren Patienten durchgeführt wurden, sollten Schwangere grundsätzlich nicht teilnehmen.

Abbruchkriterien

Abbruchkriterien für eingeschlossenen Patienten sind:

- Erreichen eines Ausschlusskriteriums
- Widerruf der Einverständniserklärung

12. Versicherung

entfällt

13. Datenschutz

Die im Rahmen der o. g. Studie erhobenen Daten und Angaben zur Krankengeschichte des Patienten werden von der Studienärztin/dem Studienarzt getrennt von den persönlichen Angaben der Patienten handschriftlich und/oder elektronisch aufgezeichnet, und zwar in pseudonymisierter Form. D. h. nur die Initialen und das Alter werden elektronisch dokumentiert, so dass dies nur der/dem Studienärztin/ Studienarzt eine Zuordnung der Krankheits- bzw. Gesundheitsdaten zu der individuellen Person ermöglicht. Soweit jedoch die im Rahmen der o. g. Studie ermittelten Daten für die Diagnose der Erkrankung bzw. der weiteren Behandlung wichtig sind, werden diese auch in die Krankenakte aufgenommen. Die Ergebnisse der o. g. Studie werden ohne Bezugsmöglichkeit auf den individuellen Patienten voraussichtlich in einer medizinischen Fachzeitschrift veröffentlicht.

Case Report Form- ARFI-SD-Studie

Patienten-Nr. ☐☐☐☐ **Pat.-Initialen:** ☐☐ **Geschlecht: w** ☐ **m** ☐

Alter: ☐☐

Untersuchungsdatum: ☐☐ **/** ☐☐ **/** 2 0 ☐☐ **:**

Datum der Szintigraphie: ☐☐ **/** ☐☐ **/** 2 0 ☐☐ **:**

Befund Szinti: ☐ kalt ☐ indifferent ☐ ***heiss (Auschlusskriterium!)***

Datum der FNA: ☐☐ **/** ☐☐ **/** 2 0 ☐☐ **:**

Befund FNA: ________________________________

Datum der OP: ☐☐ **/** ☐☐ **/** 2 0 ☐☐ **:**

Befund OP: ________________________________

MiBi Szinti: ☐ ja ☐ nein **Befund**________________________________

Patientencharakteristika:

Erstdiagnose Knoten	
Wachstum des Knoten	☐ Ja ☐ Nein
Rezidiv-Knoten	☐ Ja ☐ Nein
Vorangegangene Radiatio	☐ Ja ☐ Nein
SD-Medikation, welche	
Gewichtsverlust	☐ Ja ☐ Nein
Karzinom/Metastasen in der Anamnese	☐ Ja ☐ Nein
Seite des untersuchten Knotens	☐ rechts ☐ links
Struma oder solitärer Knoten	
Schilddrüenvolumen rechts (ml)	
Schilddrüenvolumen links (ml)	
Schilddrüenvolumen gesamt (ml)	
Echogenität der Schilddrüse	☐ hypoechogen ☐ hyper ☐ iso

Grösse des Knoten (Länge x Breite x Tiefe)	
Halosaum	☐ Ja ☐ Nein
Begrenzung des Knoten	☐ glatt ☐ unregelmäßig
Form des Knoten	☐ rund ☐ ovalär ☐ polizyklisch
Echogenität des Knotens	☐ schwächer ☐ stärker ☐ iso
Homogenität des Knotens	☐ homogen ☐ inhomogen
Makrokalzifikationen	☐ Ja ☐ Nein
Mikrokalzifikationen	☐ Ja ☐ Nein
Zystische Veränderungen	☐ Ja ☐ Nein
Power Doppler Perfusion-Knoten (1-4)	
Elastographie-Score (ES1-4)	

Patienten-Nr. □□□ □ **Pat.-Initialen:** □□

ARFI-Imaging Knoten

Zahl der Fehlmessungen			
Messung 1		Messung 6	
Messung 2		Messung 7	
Messung 3		Messung 8	
Messung 4		Messung 9	
Messung 5		Messung 10	

ARFI-Imaging gesunde Schilddrüse

Zahl der Fehlmessungen			
Messung 1		Messung 6	
Messung 2		Messung 7	
Messung 3		Messung 8	
Messung 4		Messung 9	
Messung 5		Messung 10	

Literatur

1. Reiners C, Wegscheider K, Schicha H, Theissen P, Vaupel R, Wrbitzky R, Schumm-Draeger PM. Prevalence of thyroid disorders in the working population of Germany: ultrasonography screening in 96,278 unselected employees. *Thyroid* 2004; 14: 926-32.

2. Iannuccilli JD, Cronan JJ, Monchik JM. Risk for malignancy of thyroid nodules as assessed by sonographic criteria: the need for biopsy. *J Ultrasound Med* 2004; 23: 1455-64.

3. Dietlein M, Dressler J, Grünwald F, Joseph K, Leisner B, Moser E, Reiners C, Rendl J, Schicha H, Schneider P, Schober O. Leitlinie zur Schilddrüsendiagnostik. Deutsche Gesellschaft für Nuklearmedizin e.V. 2-1-2003.
4. Hegedus L. Clinical practice. The thyroid nodule. *N Engl J Med* 2004; 351: 1764-71.

5. Hegedus L, Bonnema SJ, Bennedbaek FN. Management of simple nodular goiter: current status and future perspectives. *Endocr Rev* 2003; 24: 102-32.

6. Meller J, Becker W. The continuing importance of thyroid scintigraphy in the era of high-resolution ultrasound. *Eur J Nucl Med Mol Imaging* 2002; 29 Suppl 2: S425-S438.

7. Cooper DS, Doherty GM, Haugen BR, Kloos RT, Lee SL, Mandel SJ, Mazzaferri EL, McIver B, Sherman SI, Tuttle RM. Management guidelines for patients with thyroid nodules and differentiated thyroid cancer. *Thyroid* 2006; 16: 109-42.

8. Gharib H, Goellner JR, Johnson DA. Fine-needle aspiration cytology of the thyroid. A 12-year experience with 11,000 biopsies. *Clin Lab Med* 1993; 13: 699-709.

9. Gharib H, Goellner JR. Fine-needle aspiration biopsy of the thyroid: an appraisal. *Ann Intern Med* 1993; 118: 282-9.

10. Hegedus L. Thyroid ultrasound. *Endocrinol Metab Clin North Am* 2001; 30: 339-60.

11. Cappelli C, Castellano M, Pirola I, Cumetti D, Agosti B, Gandossi E, Agabiti RE. The predictive value of ultrasound findings in the management of thyroid nodules. *QJM* 2007; 100: 29-35.

12. Wienke JR, Chong WK, Fielding JR, Zou KH, Mittelstaedt CA. Sonographic features of benign thyroid nodules: interobserver reliability and overlap with malignancy. *J Ultrasound Med* 2003; 22: 1027-31.

13. Koike E, Noguchi S, Yamashita H, Murakami T, Ohshima A, Kawamoto H, Yamashita H. Ultrasonographic characteristics of thyroid nodules: prediction of malignancy. *Arch Surg* 2001; 136: 334-7.

14. Cappelli C, Castellano M, Pirola I, Gandossi E, De Martino E, Cumetti D, Agosti B, Rosei EA. Thyroid nodule shape suggests malignancy. *Eur J Endocrinol* 2006; 155: 27-31.

15. Cappelli C, Pirola I, Cumetti D, Micheletti L, Tironi A, Gandossi E, Martino E, Cherubini L, Agosti B, Castellano M, Mattanza C, Rosei EA. Is the anteroposterior and transverse diameter ratio of nonpalpable thyroid nodules a sonographic criteria for recommending fine-needle aspiration cytology? *Clin Endocrinol (Oxf)* 2005; 63: 689-93.

16. Iannuccilli JD, Cronan JJ, Monchik JM. Risk for malignancy of thyroid nodules as assessed by sonographic criteria: the need for biopsy. *J Ultrasound Med* 2004; 23: 1455-64.

17. Kim EK, Park CS, Chung WY, Oh KK, Kim DI, Lee JT, Yoo HS. New sonographic criteria for recommending fine-needle aspiration biopsy of nonpalpable solid nodules of the thyroid. *AJR Am J Roentgenol* 2002; 178: 687-91.

18. Appetecchia M, Solivetti FM. The association of colour flow Doppler sonography and conventional ultrasonography improves the diagnosis of thyroid carcinoma. *Horm Res* 2006; 66: 249-56.

19. Papini E, Guglielmi R, Bianchini A, Crescenzi A, Taccogna S, Nardi F, Panunzi C, Rinaldi R, Toscano V, Pacella CM. Risk of malignancy in nonpalpable thyroid nodules: predictive value of ultrasound and color-Doppler features. *J Clin Endocrinol Metab* 2002; 87: 1941-6.

20. Fukunari N, Nagahama M, Sugino K, Mimura T, Ito K, Ito K. Clinical evaluation of color Doppler imaging for the differential diagnosis of thyroid follicular lesions. *World J Surg* 2004; 28: 1261-5.

21. Frates MC, Benson CB, Doubilet PM, Cibas ES, Marqusee E. Can color Doppler sonography aid in the prediction of malignancy of thyroid nodules? *J Ultrasound Med* 2003; 22: 127-31.

22. De Nicola H, Szejnfeld J, Logullo AF, Wolosker AM, Souza LR, Chiferi V, Jr. Flow pattern and vascular resistive index as predictors of malignancy risk in thyroid follicular neoplasms. *J Ultrasound Med* 2005; 24: 897-904.

23. Miyakawa M, Onoda N, Etoh M, Fukuda I, Takano K, Okamoto T, Obara T. Diagnosis of thyroid follicular carcinoma by the vascular pattern and velocimetric parameters using high resolution pulsed and power Doppler ultrasonography. *Endocr J* 2005; 52: 207-12.

24. Hegedus L. Clinical practice. The thyroid nodule. *N Engl J Med* 2004; 351: 1764-71.

25. Frey H. Realtime elastography. A new ultrasound procedure for the reconstruction of tissue elasticity. *Radiologe* 2003; 43: 850-5.

26. Asteria C, Giovanardi A, Pizzocaro A, Cozzaglio L, Morabito A, Somalvico F, Zoppo A. US-elastography in the differential diagnosis of benign and malignant thyroid nodules. *Thyroid* 2008; 18: 523-31.

27. Dighe M, Bae U, Richardson ML, Dubinsky TJ, Minoshima S, Kim Y. Differential diagnosis of thyroid nodules with US elastography using carotid artery pulsation. *Radiology* 2008; 248: 662-9.

28. Lyshchik A, Higashi T, Asato R, Tanaka S, Ito J, Mai JJ, Pellot-Barakat C, Insana MF, Brill AB, Saga T, Hiraoka M, Togashi K. Thyroid gland tumor diagnosis at US Elastography. *Radiology* 2005; 237: 202-11.

29. Rubaltelli L, Corradin S, Dorigo A, Stabilito M, Tregnaghi A, Borsato S, Stramare R. Differential Diagnosis of Benign and Malignant Thyroid Nodules at Elastosonography. *Ultraschall Med* 2008.

30. Rago T, Santini F, Scutari M, Pinchera A, Vitti P. Elastography: new developments in ultrasound for predicting malignancy in thyroid nodules. *J Clin Endocrinol Metab* 2007; 92: 2917-22.

31. Friedrich-Rust M, Wunder K, Kriener S, Sotoudeh F, Richter S, Bojunga J, Herrmann E, Poynard T, Dietrich CF, Vermehren J, Zeuzem S, Sarrazin C. Liver fibrosis in viral hepatitis: noninvasive assessment with acoustic radiation force impulse imaging versus transient elastography. *Radiology* 2009; 252: 595-604.
